# Birth Rate in France

**Published:** 1883-01

**Authors:** 


THE average birth rate per annum in France for the period between 1872 and 1880 has been calculated to be one birth for 37 inhabitants, which is by far the lowest birth rate in Europe. For the different countries the birth rate is as follows: Russia, one birth for 20 inhabitants; Germany, one birth for 25; Austro-Hungary, one birth for 26; England, one birth for 27; Italy, one birth for 27; Spain, one birth for 28; France, one birth for 37. If the yearly number of births for any thousand inhabitants be calculated, we have precisely the same result. We have in France, 26 births per 1,000; Belgium, 32; England, 35; Austria, 38; Prussia, 38; and Russia, 50.

THE known attempts on Louis Phillippe's life were as follows: Bergeon, on the Pont Royal, December, 1832; Fieschi, infernal machine, Boulevard, July, 1835, Alibaud, court of the Tuileries, June 1838; Meunier, Quai des Tuileries, December, 1836; Champion, an abortive infernal machine, Qui de la Conference, 1837; Darmés, near the Pont de la Concorde, October, 1840; Quenessit, who shot at the three princes, September, 1841; Lecomte, Fontainebleau, August, 1848; Henri, on the balcony of the Tuileries, July, 1847. It should be cheering to uneasy crown wearers to reflect that the object of these attacks escaped scatheless, and died in the eighties, in the peaceful seclusion of a very luxurious English country house.

